# The regulatory role of aberrant Phosphatase and Tensin Homologue and Liver Kinase B1 on AKT/mTOR/c-Myc axis in pancreatic neuroendocrine tumors

**DOI:** 10.18632/oncotarget.20956

**Published:** 2017-09-16

**Authors:** Tsung-Ming Chang, Yan-Shen Shan, Pei-Yi Chu, Shih Sheng Jiang, Wen-Chun Hung, Yu-Lin Chen, Hsiu-Chi Tu, Hui-You Lin, Hui-Jen Tsai, Li-Tzong Chen

**Affiliations:** ^1^ National Institute of Cancer Research, National Health Research Institutes, Tainan, Taiwan; ^2^ Department of Surgery, National Cheng Kung University Hospital, Tainan, Taiwan; ^3^ Institute of Clinical Medicine, National Cheng Kung University, Tainan, Taiwan; ^4^ Department of Pathology, Show Chwan Memorial Hospital, Changhua, Taiwan; ^5^ School of Medicine, College of Medicine, Fu Jen Catholic University, New Taipei City, Taiwan; ^6^ Department of Internal Medicine, National Cheng Kung University Hospital, Tainan, Taiwan; ^7^ Department of Internal Medicine, Kaohsiung Medical University Hospital, Kaohsiung, Taiwan; ^8^ Institute of Molecular Medicine, National Cheng Kung University, Tainan, Taiwan

**Keywords:** pancreatic neuroendocrine tumor, PTEN, LKB1, mTOR, c-Myc

## Abstract

Pancreatic neuroendocrine tumor (pNET) is an uncommon type of pancreatic neoplasm. Low Phosphatase and Tensin Homologue (PTEN) expression and activation of the mechanistic target of rapamycin (mTOR) pathway have been noted in pNETs, and the former is associated with poor survival in pNET patients. Based on the results of the RADIANT-3 study, everolimus, an oral mTOR inhibitor, has been approved to treat advanced pNETs. However, the exact regulatory mechanism for the mTOR pathway in pNETs remains largely unknown. PTEN and liver kinase B1 (LKB1) are well-known for their regulatory role in the mTOR pathway. We evaluated the expression of PTEN and LKB1 in 21 pNET patients, and low PTEN and LKB1 expression levels were noted in 48% and 24% of the patients, respectively. Loss of PTEN and LKB1 synergistically promoted cell proliferation of pNET, attenuated the sensitivity of cells to mTOR inhibitors and enhanced c-Myc expression, which back-regulated PTEN, AKT, mTOR and its downstream effectors. For pNET cells with low expression levels of PTEN and LKB1, silencing the expression of c-Myc by shRNA reduced their proliferative rate, while adding either c-Myc inhibitor or AMP-activated protein kinase activator reversed their resistance to mTOR inhibitors *in vitro* and *in vivo*. Furthermore, high c-Myc expression was subsequently identified in 81% of pNETs, suggesting that up-regulation of c-Myc expression in pNETs may occur through PTEN/LKB1-dependent and PTEN/LKB1-independent regulation. The results delineated the regulation of PTEN and LKB1 on the AKT/mTOR/c-Myc axis and suggested that both c-Myc and mTOR are potential therapeutic targets for pNET.

## INTRODUCTION

Pancreatic neuroendocrine tumor (pNET) is a pancreatic neoplasm that expresses neuroendocrine markers. There has been a worldwide increase in the incidence of NETs [1–3]. Activation of the mechanistic target of rapamycin (mTOR) pathway in pNET has been observed by several researchers either using gene expression array or immunohistochemistry in recent years [4–6]. Missiaglia et al. showed that mTOR inhibitors successfully inhibit the proliferation of pNET cell lines [4]. Everolimus (RAD001), a mTOR inhibitor, prolongs the median progression-free survival for advanced pNET to 11 months versus 4.6 months in patients taking placebo as indicated by a phase III study [7]. Thus, everolimus is currently used to treat metastatic pNET.

Phosphatase and tensin homologue (*PTEN*) was identified to be frequently disrupted in multiple sporadic tumor types in 1997 [8, 9]. The generation of *PTEN* knockout mice demonstrated the essential tumor suppressive role of PTEN in multiple tissue types. PTEN dephosphorylates phosphatidylinositol-3,4,5-triphosphate (PtdIns(3,4,5)P_3_), which potently activates 3-phosphoinositide-dependent kinase (PDK) and AKT with subsequent activation of mTOR and its downstream targets p70 ribosomal protein S6 kinase (S6K) and eukaryotic translation-initiation factor 4E (eIF4E)-binding protein 1 (4EBP1). Therefore, PTEN is a potent inhibitor of the PI3K-AKT-mTOR pathway, which stimulates cell growth and survival [10]. Low expression of PTEN is correlated with a shorter disease-free survival and overall survival of pNET [4, 11]. In preclinical studies, PTEN deficiency or loss of PTEN is associated with responsiveness to mTOR inhibitors in endometrial cancer, Ewing sarcoma, and breast cancer [12–14]. The regulation of the mTOR pathway by PTEN and the sensitivity of pNET to mTOR inhibitors remain unclear.

Liver kinase B1 (LKB1) is a tumor suppressor. Defective LKB1 is responsible for the inherited cancer disorder, Peutz-Jeghers syndrome, and is found in various sporadic cancers, such as lung and cervical cancers. LKB1 is the key upstream activator of AMP-activated protein kinase (AMPK), which is the central metabolic switch in eukaryotes to govern glucose and lipid metabolism in response to changes in nutrients and intracellular energy. The mTOR pathway is one of the major growth regulatory pathways controlled by LKB1-AMPK [15]. Therefore, suppression of the mTOR pathway by an AMPK activator has been evaluated for therapeutics in cancers. Reduced expression of LKB1 is common in high grade neuroendocrine carcinoma of lung and less common in pulmonary carcinoids [16]. However, the expression of LKB1 and its role in the regulation of mTOR in pNET remain unknown.

Although mTOR activation in pNET is well known, regulation of the mTOR pathway in pNET is not well understood. In this study, we evaluated PTEN and LKB1 expression in pNETs by immunohistochemistry, and we investigated the effect of PTEN and LKB1 on the regulation of the mTOR pathway in pNET cell lines via gene overexpression or knockdown. In addition, we evaluated the effect of PTEN and LKB1 on the sensitivity of pNET cells to mTOR inhibitors.

## RESULTS

### PTEN and LKB1 expression in pNET patients

**Figure 1:**
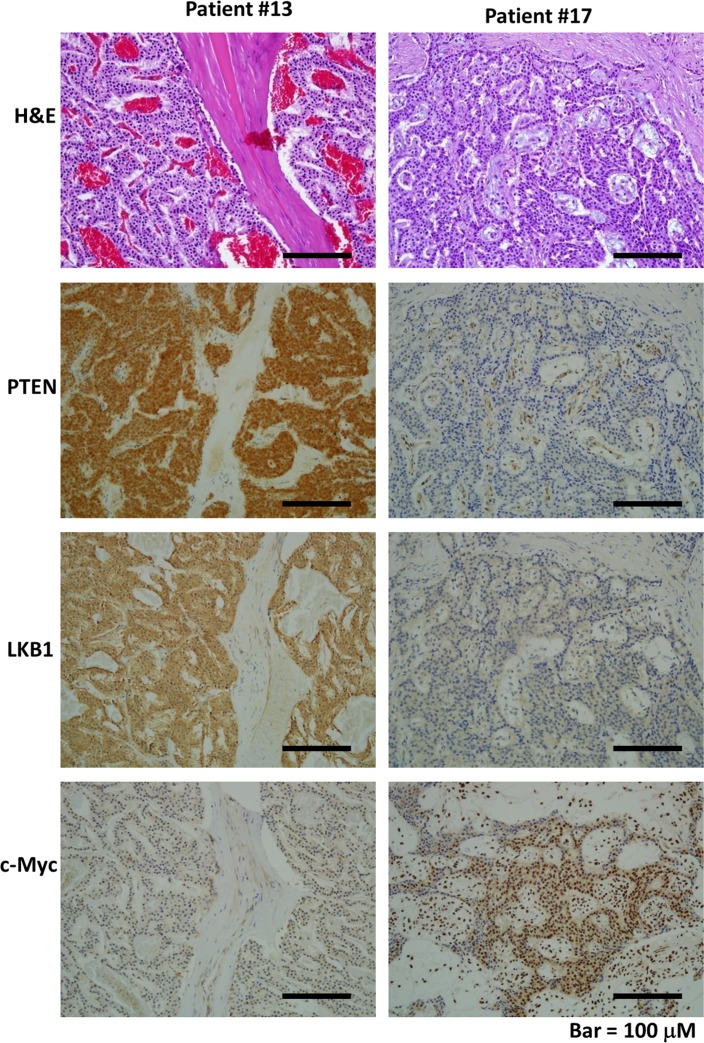
The hematoxylin and eosin (H&E) and immunohistochemical staining patterns for LKB1, PTEN, and c-Myc in the PNET tumor cells are shown (200×) Patient #13 had strong expression for PTEN and LKB1 in tumor cells but low expression for c-Myc in tumor cells. Patient #17 had weak expression for PTEN and LKB1 but strong expression for c-Myc in tumor cells.

We evaluated PTEN and LKB1 expression in tumor samples from pNET patients by immunohistochemistry. The expression status of the proteins was scored from 0 to 3+ according to the strength of the staining as follows: 0 and 1+ were classified as low expression; and 2+ and 3+ were classified as high expression. PTEN and LKB1 staining was present in the cytoplasm of pNET tumors. The expression of PTEN and LKB1 in two cases is shown in Figure 1. The demographics and expression status of PTEN and LKB1 of the patients are listed in Supplementary Table 1. Among the 21 pNET patients, 10 had low PTEN expression, and 5 had low LKB1 expression. Three patients had low expression of both PTEN and LKB1. There was no association of PTEN and LKB1expression with the sex, age, grade, stage or survival of the patients (Supplementary Table 2).

### PTEN affects cell proliferation and regulates the AKT/mTOR pathway of pNET cells

**Figure 2:**
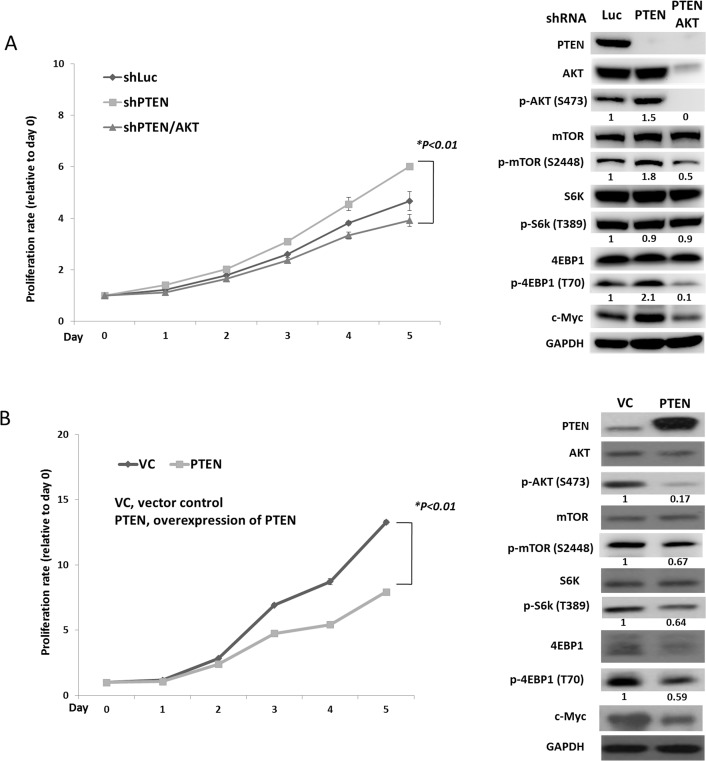
PTEN affects cell proliferation and regulates the AKT/mTOR pathway of pNET cells (**A**) The cell proliferative curves of QGP-1 cells with (QGP-1/shPTEN) or without (QGP-1/shLuc) knockdown of PTEN and knockdown of PTEN and AKT (QGP-1/shPTEN/AKT) as well as the protein expression of PTEN, c-Myc and total and phosphorylated forms of AKT, mTOR, S6K and 4EBP1 in QGP-1/shLuc, QGP-1/shPTEN and QGP-1/shPTEN/AKT cells. ^*^, QGP-1/shPTEN vs. QGP-1/shPTEN/AKT, *P* < 0.01. The ratio of protein expression of phosphorylated AKT, mTOR, S6K and 4EBP1 of QGP-1/shPTEN and QGP-1/shPTEN/AKT relative to QGP-1/shLuc is shown below the western blot of each protein. The protein expression was adjusted to its internal control GAPDH. (**B**) The cell proliferative curves of QGP-1 cells with (QGP-1/PTEN) or without (QGP-1/VC) PTEN as well as the protein expression of PTEN, total and phosphorylated forms of AKT, mTOR, S6K and 4EBP1 in both cells. ^*^, QGP-1/VC vs. QGP-1/PTEN, *P* < 0.01. The ratio of protein expression of phosphorylated AKT, mTOR, S6K and 4EBP1 of QGP-1/PTEN relative to QGP-1/VC is shown below the western blot of each protein. The protein expression was adjusted to its internal control GAPDH.

To evaluate the effect of PTEN on pNET, we infected the human pNET cell line, QGP-1, with a lentiviral vector containing a PTEN shRNA (QGP-1/shPTEN). Cells infected with a lentiviral vector containing a luciferase shRNA (QGP-1/shLuc) were used as a control. The cell proliferative rate was slightly higher in QGP-1/shPTEN cells than in QGP-1/shLuc cells as shown in Figure 2A. The phosphorylation of AKT, mTOR and 4EBP1 in QGP-1/shPTEN cells was enhanced as shown in Figure 2A. In contrast, when PTEN was overexpressed in QGP-1 cells using a lentiviral pCMV Flag WT-PTEN vector, the cell proliferative rate was significantly reduced as compared to QGP-1 cells infected with a vector control as shown in Figure 2B. The phosphorylation of AKT, S6K and 4EBP1 was reduced markedly by overexpressing PTEN in QGP-1 cells as shown in Figure 2B. When AKT was knocked down in QGP-1/shPTEN cells, the enhanced phosphorylation of 4EBP1 in QGP-1/shPTEN cells was suppressed, and the increased proliferative rate of QGP-1/shPTEN cells was decreased to a level similar to that of QGP-1/shLuc cells as shown in Figure 2A. The results demonstrated the involvement of the PTEN/AKT/mTOR axis in the proliferation of pNET cells.

### PTEN and LKB1 loss synergistically enhances the activation of AKT/mTOR pathway, promotes the proliferation of pNET cell lines and confers the attenuated sensitivity of pNET cells to mTOR inhibitors

**Figure 3:**
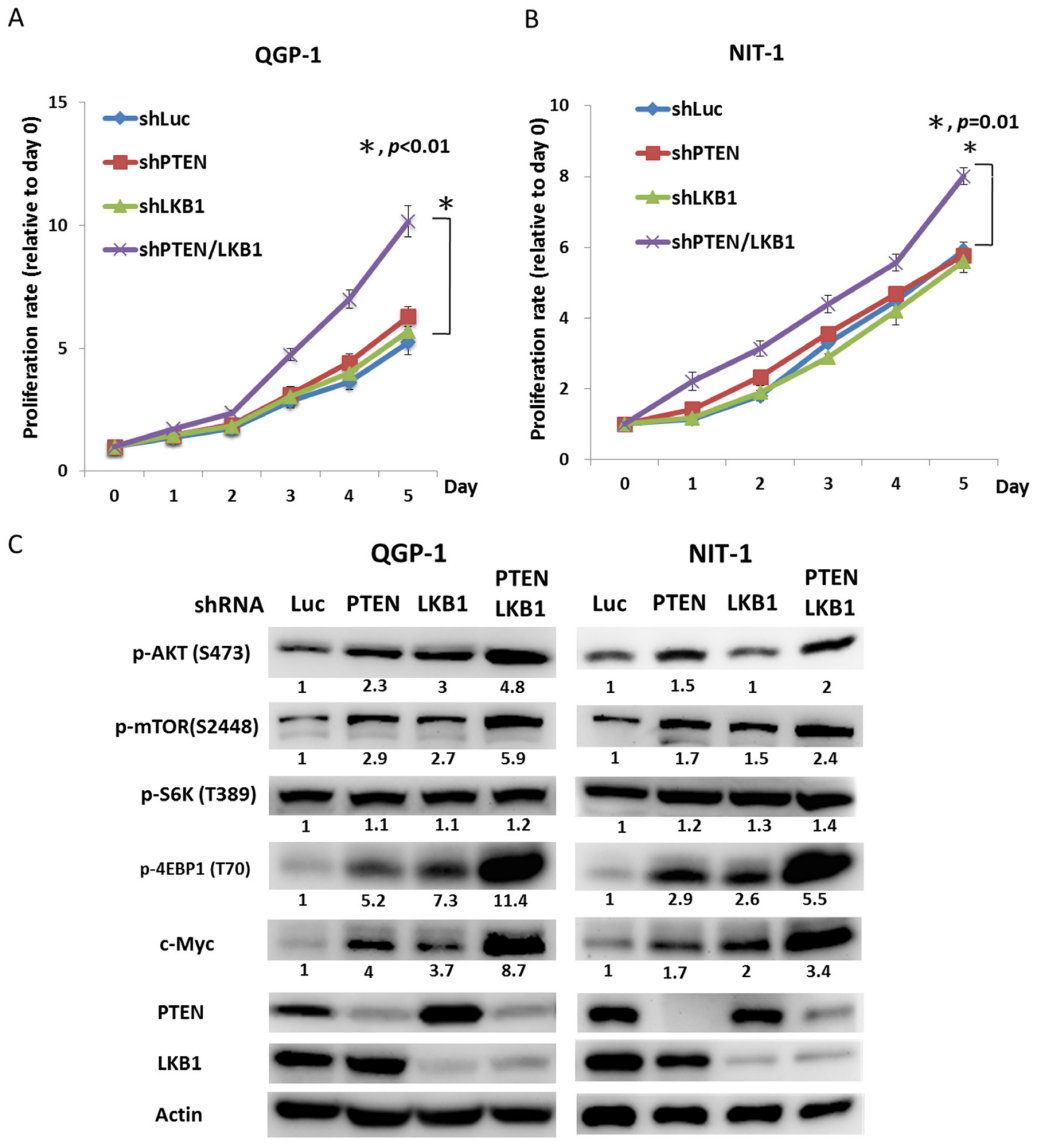
PTEN and LKB1 synergistically enhance the activation of the AKT/mTOR pathway and promote the proliferation of pNET cell lines (**A**) The cell proliferative rates of QGP-1 cells without (QGP-1/shLuc) or with knockdown of PTEN (QGP-1/shPTEN), LKB1 (QGP-1/shLKB1) and both PTEN and LKB1 (QGP-1/shPTEN/LKB1). ^*^, QGP-1/shLuc vs. QGP-1/shPTEN/LKB1, *P* < 0.01 (**B**) The cell proliferative rates of NIT-1 cells without (NIT-1/shLuc) or with knockdown of PTEN (NIT-1/shPTEN), LKB1 (QGP-1/shLKB1) and both PTEN and LKB1 (QGP-1/shPTEN/LKB1). ^*^, NIT-1/shLuc vs. NIT-1/shPTEN/LKB1, *P* = 0.01 (**C**) The phosphorylated protein expression of AKT, mTOR, S6K and 4EBP1 as well as the protein expression of c-Myc, PTEN and LKB1 in QGP-1 and NIT-1 cells without or with knockdown of PTEN, LKB1 and both PTEN and LKB1 by western blot. Actin was used as the internal control.

**Figure 4:**
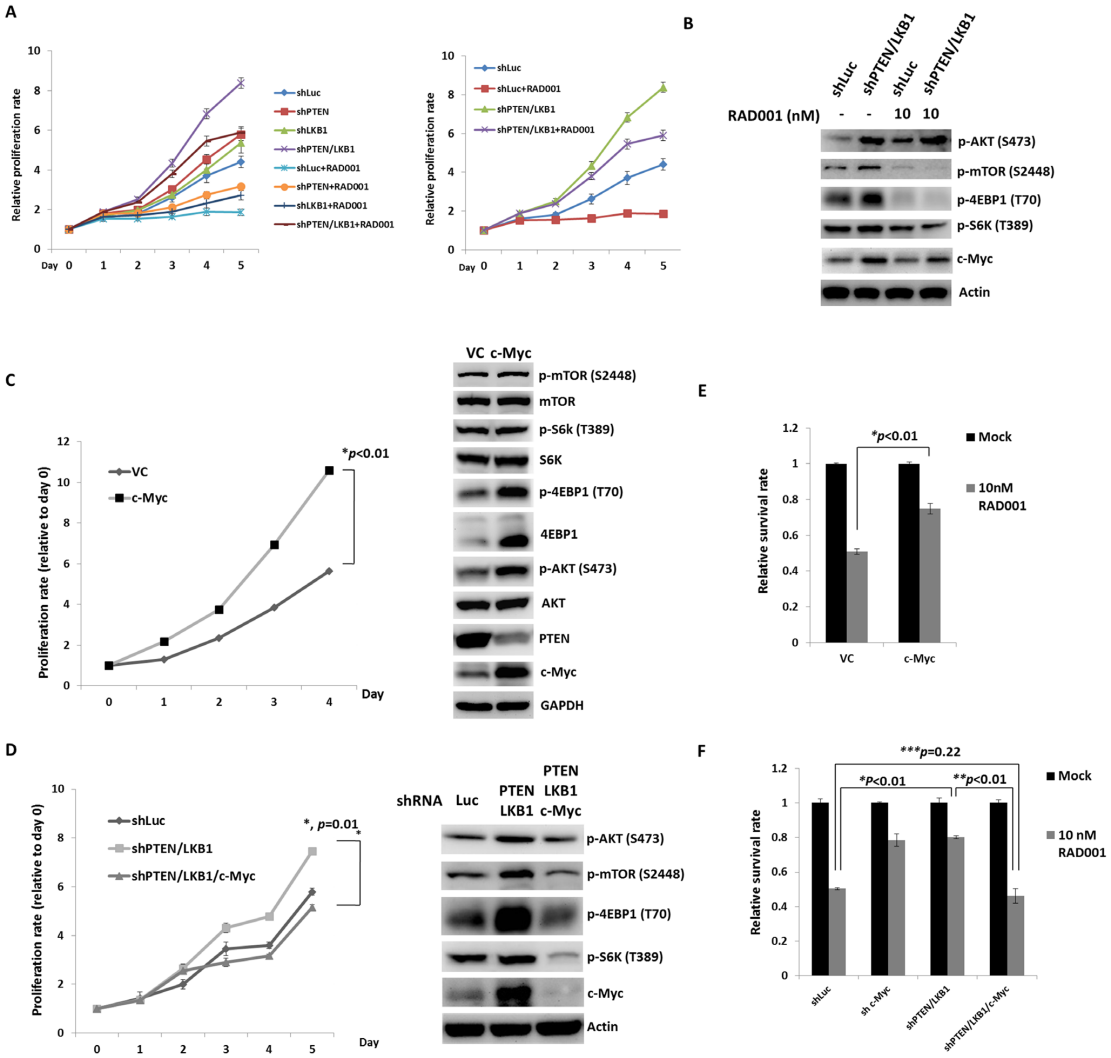
PTEN and LKB1 loss confers the attenuated sensitivity of pNET cells to mTOR inhibitor via regulation of AKT/mTOR/c-Myc axis (**A**) The proliferative curves of 4 kinds of QGP-1 cells, QGP-1/shLuc, QGP-1/shPTEN, QGP-1/shLKB1, QGP-1/shPTEN/LKB1 treated without (mock) or with 10 nM of RAD001 for 5 days. (**B**) The expression of c-Myc and phosphorylated form of AKT, mTOR, S6K and 4EBP1 in QGP-1 cells without or with knockdown of both PTEN and LKB1 with or without exposure to 10 nM of RAD001 for 48 hours by western blot. (**C**) The proliferative rate of QGP-1 cells with (QGP-1/c-Myc) or without (QGP-1/VC) overexpression of c-Myc and the expression of c-Myc, PTEN and total and phosphorylated AKT, mTOR, S6k and 4EBP1. (**D**) The cell proliferative rate of QGP-1 cells with double knockdown of PTEN and LKB1 and triple knockdown of PTEN, LKB1 and c-Myc compared with the proliferative rate of QGP-1/shLuc cells and the protein expression of c-Myc and phosphorylated form of AKT, mTOR, S6K and 4EBP1 in QGP-1/shLuc, QGP-1/shPTEN/LKB1 and QGP-1/shPTEN/LKB1/c-Myc cells. (**E**) The survival rate of QGP-1/VC and QGP-1/c-Myc treated with or without 10 nM of RAD001 for 72 hours. ^*^, QGP-1/VC treated with RAD001 vs. QGP-1/c-Myc treated with RAD001, *P* < 0.01 (**F**) The survival rate of QGP-1/shLuc, QGP-1/shPTEN/LKB1 and QGP-1/shPTEN/LKB1/c-Myc treated with or without RAD001 for 72 hours. The survival of QGP-1/shPTEN/LKB1 treated with RAD001 was better than that of QGP-1/shLuc cells treated with RAD001. When the QGP-1/shPTEN/LKB1 cells were knocked down with c-Myc, the survival of QGP-1/shPTEN/LKB1/c-Myc treated with RAD001 was similar to that of QGP-1/shLuc treated with RAD001. ^*^, QGP-1/shLuc treated with RAD001 vs. QGP-1/shPTEN/LKB1 treated with RAD001, *P* < 0.01; ^**^, QGP-1/shPTEN/LKB1 treated with RAD001 vs. QGP-1/shPTEN/LKB1/c-Myc treated with RAD001, *P* < 0.01; ^***^, QGP-1/shLuc treated with RAD001 vs. QGP-1/shPTEN/LKB1/c-Myc treated with RAD001, *P* = 0.22.

To evaluate the effect of LKB1 on cell proliferation and regulation of the mTOR pathway in pNET cells, we infected QGP-1 cells with a lentiviral vector containing LKB1 shRNA (QGP-1/shLKB1). Cell proliferation was not significantly different from that of QGP-1/shLuc cells as shown in Figure 3A, but the phosphorylation of AKT, mTOR, and 4EBP1 was enhanced by knockdown of LKB1 as shown in Figure 3C. When both PTEN and LKB1 in QGP-1 cells were knocked down (QGP-1/shPTEN/LKB1), the phosphorylation of AKT, mTOR and 4EBP1 was further enhanced as shown in Figure 3C, and cell proliferation was also significantly increased as shown in Figure 3A. The effects of LKB1 and PTEN on cell proliferation and the mTOR pathway were also verified in the murine pNET cell line, NIT-1, as shown in Figure 3B and 3C. Because enhanced activation of mTOR and its downstream effectors was noted in QGP-1/shPTEN/LKB1 cells, we further evaluated if the sensitivity of QGP-1 cells to mTOR inhibitors was affected by PTEN and/or LKB1 loss. Figure 4A shows the proliferative rate of QGP-1/shLuc, QGP-1/shPTEN, QGP-1/shLKB1 and QGP-1/shPTEN/LKB1 cells treated with or without 10nM of RAD001 for 5 days. QGP-1/shPTEN/LKB1 cells had the highest proliferative rate compared to the other three cells (Figure 4A, left). The proliferative curves of QGP-1/shLuc and QGP-1/PTEN/LKB1 cells treated with or without RAD001 are displayed in the right side of Figure 4A. The proliferative rate of QGP-1/shPTEN/LKB1 cells treated with RAD001 was higher than that of QGP-1/shLuc cells treated with RAD001 for 5 days. Because the cells proliferated with RAD001 treatment, these results suggested that PTEN and LKB1 loss synergistically promotes proliferation of pNET cells and attenuates the sensitivity of pNET cells to mTOR inhibitors via a cytostatic effect.

### c-Myc is the downstream target of mTOR and confers the enhanced proliferation of pNET cells and attenuates the sensitivity of pNET cells to a mTOR inhibitor via feedback regulation of the PTEN/AKT/mTOR axis

Because the sensitivity of pNET cells to mTOR inhibitors was decreased by PTEN and LKB1 loss, we evaluated the status of mTOR and its downstream signals in QGP-1/shPTEN/LKB1 and QGP-1/shLuc cells treated with or without RAD001 by western blot analysis. RAD001 suppressed the phosphorylation of mTOR, S6K, and 4EBP1 in QGP-1/shPTEN/LKB1 and QGP-1/shLuc cells as shown in Figure 4B. However, the effective dephosphorylation of mTOR, S6K and 4EBP1 in QGP-1/shPTEN/LKB1 cells did not explain the attenuated sensitivity of QGP-1/shPTEN/LKB1 cells to RAD001. Because c-Myc is a downstream target of mTOR in many cancers, we evaluated c-Myc expression in the cells and found that c-Myc expression was increased in QGP-1/shPTEN and QGP-1/shLKB1 cells and was further enhanced in QGP-1/shPTEN/LKB1 cells as compared to QGP-1/shLuc cells as shown in Figure 3C. This up-regulation of c-Myc occurred at a transcriptional level because QRT-PCR showed increased c-Myc mRNA levels in QGP-1/shPTEN, QGP-1/shLKB1 and QGP-1/shPTEN/LKB1 cells compared to QGP-1/shLuc cells as shown in Supplementary Figure 1.The up-regulation of c-Myc by PTEN and/or LKB1 loss was also observed in NIT-1 cells as shown in Figure 3C. Figure 4B shows that the reduction in c-Myc levels in QGP-1/shPTEN/LKB1 cells was less extensive compared to that in QGP-1/shLuc cells treated with RAD001. These results suggested that up-regulated c-Myc may have been responsible for the attenuated sensitivity of QGP-1/shPTEN/LKB1 cells to RAD001 as well as the rapid cell proliferation. To verify this hypothesis, we overexpressed c-Myc by introducing pLM-mCerulean-cMyc into QGP-1 cells (QGP-1/c-Myc), and we evaluated cell proliferation and the AKT/mTOR pathway. Figure 4C shows that QGP-1/c-Myc cells had a higher proliferative rate and markedly enhanced phosphorylation of AKT and 4EBP1 compared to QGP-1 cells infected with vector control (QGP-1/VC). Intriguingly, PTEN expression was reduced in QGP-1/c-Myc cells compared to that in QGP-1/VC cells. In contrast, the increased proliferative rate as well as the enhanced phosphorylation of AKT, mTOR, and 4EBP1 in QGP-1/shPTEN/LKB1 cells were suppressed by knockdown of c-Myc (QGP-1/shPTEN/LKB1/c-Myc) as shown in Figure 4D. When QGP-1/c-Myc and QGP-1/VC cells were treated with 10 nM RAD001 for 72 hours, the survival of QGP-1/c-Myc cells was better than QGP-1/VC cells as shown in Figure 4E. In contrast, when QGP-1/shPTEN/LKB1/c-Myc cells were treated with RAD001, the sensitivity of QGP-1/shPTEN/LKB1/c-Myc cells to RAD001 was similar to the sensitivity of QGP-1/shLuc to RAD001 cells as shown in Figure 4F. The increased resistance to RAD001 resulting from knockdown of PTEN and LKB1 was reversed by knockdown of c-Myc in QGP-1/shPTEN/LKB1 cells. These results indicated that c-Myc is associated with the proliferation of pNET cells and is responsible for the resistance of pNET cells to mTOR inhibitors. However, the enhanced expression of c-Myc resulting from PTEN/LKB1 loss in pNET cells was associated with mTOR-dependent and mTOR-independent regulation. Figure 2A shows that the increased c-Myc expression in QGP-1/shPTEN cells was reduced by knockdown of AKT. In addition, RAD001 inhibited the activity of mTOR as demonstrated by dephosphorylation of S6K and 4EBP1 and reduced c-Myc levels in QGP-1 cells as shown in 4B. These results indicated that c-Myc is downstream of AKT/mTOR in pNET cells. However, the less effective suppression of c-Myc in QGP-1/shPTEN/LKB1 cells compared to that in QGP-1/shLuc cells by RAD001 suggested that the regulation of c-Myc occurs through another mTOR-independent pathway. Moreover, the decreased expression of PTEN and the increased phosphorylation of AKT and 4EBP1 resulting from c-Myc overexpression in QGP-1 cells suggested that c-Myc back regulates the PTEN/AKT/mTOR pathway in pNET cells. In addition, enhanced expression of PTEN and decreased phosphorylation of AKT resulted from knockdown of c-Myc in QGP-1 cells as shown in Supplementary Figure 2. These results indicated the capability of c-Myc to confer resistance of pNET cells to mTOR inhibitors as well as to delineate the regulation of the AKT/mTOR/c-Myc axis by PTEN and LKB1 and the back regulation of PTEN/AKT/mTOR by c-Myc.

### Regulation of mTOR pathway by metformin or targeting c-Myc by c-Myc inhibitor reverses the attenuated sensitivity of pNET cells lacking PTEN/LKB1 to mTOR inhibitors

**Figure 5:**
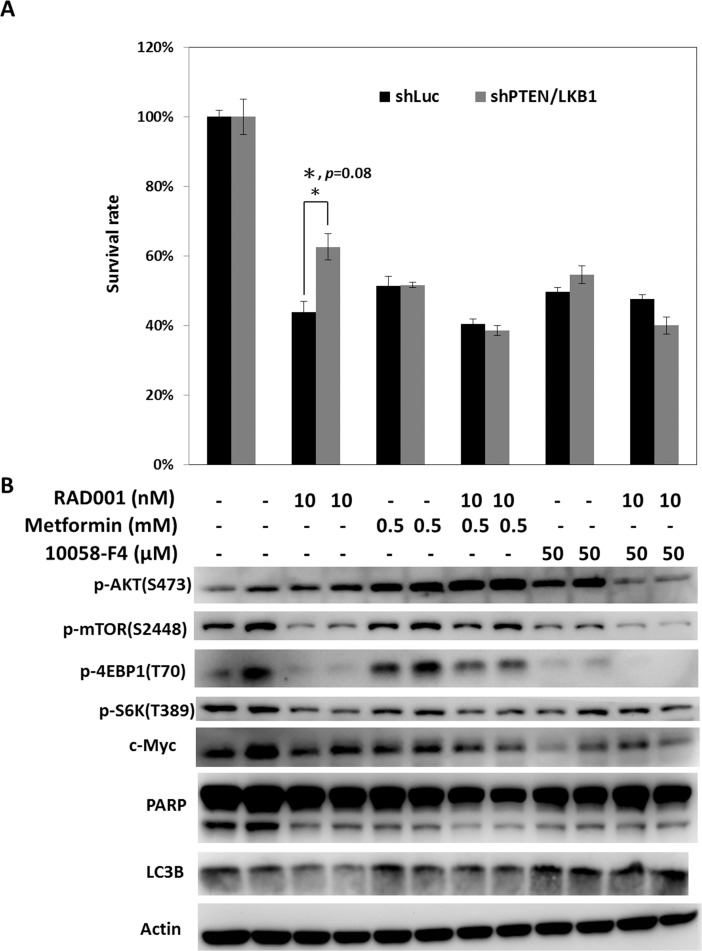
Regulation of mTOR pathway by metformin or targeting c-Myc by c-Myc inhibitor reverses the attenuated sensitivity of pNET cells with PTEN/LKB1 loss to mTOR inhibitor (**A**) The survival rate of QGP-1/shLuc and QGP-1/shPTEN/LKB1 cells treated with indicated dose of RAD001, metformin, 10058-F4 and combination of the agents for 72 hours. The QGP-1/shPTEN/LKB1 cells had significant better survival than QGP-1/shLuc cells when treated with RAD001 alone. The survival rates between QGP-1/shLuc and QGP-1/shPTEN/LKB1 cells treated with metforim or 10058-F4 alone or RAD001 combined with metform or 10058-F4 were not significantly different. ^*^, QGP-1/shLuc treated with RAD001 vs. QGP-1/shPTEN/LKB1 treated with RAD001, *P* = 0.08 (**B**) The expression of c-Myc, PARP, LC3B and phosphorylated form of AKT, mTOR,, S6K and 4EBP1 in QGP-1/shLuc and QGP-1/shPTEN/LKB1 cells exposed to the indicated dose of RAD001, metformin, 10058-F4 and combination of the agents.

As enhanced c-Myc expression resulting from PTEN and LKB1 loss was associated with the resistance of pNET cells to mTOR inhibitors, targeting c-Myc is a potential therapeutic approach for pNET. Because c-Myc can be regulated by the mTOR pathway, drugs that modulate mTOR activity, such as an AMPK activator, are also potential therapeutic agents for pNET. We treated QGP-1/shPTEN/LKB1 and QGP-1/shLuc cells with RAD001, metformin, 10058-F4 (c-Myc inhibitor), and combination of reagents at the indicated dose for 72 hours, and we measured the survival rate of the cells as shown in Figure 5A. The survival of QGP-1/shPTEN/LKB1 and QGP-1/shLuc cells was reduced to a similar extent by metformin or 10058-F4 alone, but the survival of QGP-1/shPTEN/LKB1 cells was better than that of QGP-1/shLuc cells when treated with RAD001 alone. Combination of RAD001 with metformin or 10058-F4 further decreased the survival of both cells and reversed the attenuated sensitivity of QGP-1/shPTEN/LKB1 cells to RAD001 as shown in Figure 5A. The c-Myc levels and phosphorylation levels of mTOR, 4EBP1, and S6K were decreased by RAD001 in both QGP-1/shPTEN/LKB1 and QGP-1/shLuc cells as shown in Figure 5B. Increased phosphorylation of AKT resulting from RAD001 treatment was found in both cells. When the cells were treated with metformin alone, the c-Myc levels and phosphorylation levels of 4EBP1 and S6K in QGP-1/shPTEN/LKB1 and QGP-1/shLuc cells were slightly reduced. The combination of metformin and RAD001 significantly reduced the phosphorylation of S6K but not that of mTOR and 4EBP1 in both cells to a similar extent as that by RAD001. However, the c-Myc expression in both QGP-1/shLuc and QGP-1/shPTEN/LKB1 cells was reduced to a greater extent after treatment with the combination of metformin and RAD001 compared to RAD001 alone. Compared to metformin alone, treatment with 10058-F4 alone induced greater suppression of c-Myc and phosphorylation of mTOR, 4EBP1, and S6K in both cells. When the cells were treated with RAD001 and 10058-F4, c-Myc levels and phosphorylation levels of mTOR, 4EBP1, and S6K were reduced to a greater extent than by treatment with RAD001 or 10058-F4 alone. In addition, the RAD001-induced enhanced phosphorylation of AKT in QGP-1/shLuc and QGP-1/shPTEN/LKB1 cells was suppressed by treatment with RAD001 and 10058-F4. PARP cleavage and LC3B were not significantly different or induced by RAD001, metformin, 10058-F4 or combination of the drugs. These results suggested that these agents inhibit cell proliferation but do not induce apoptosis or autophagy. These results also demonstrated that the combination of RAD001 with metformin or 10058-F4 may enhance the anti-proliferative effect in pNET cells and reverse the attenuated sensitivity of pNET cells to mTOR inhibitors caused by PTEN and LKB1 loss via a cytostatic effect.

### Synergistic loss of PTEN and LKB1 promotes tumor growth of pNET and combination of c-Myc inhibitor or metformin enhances the inhibition of pNET tumor growth induced by RAD001

**Figure 6:**
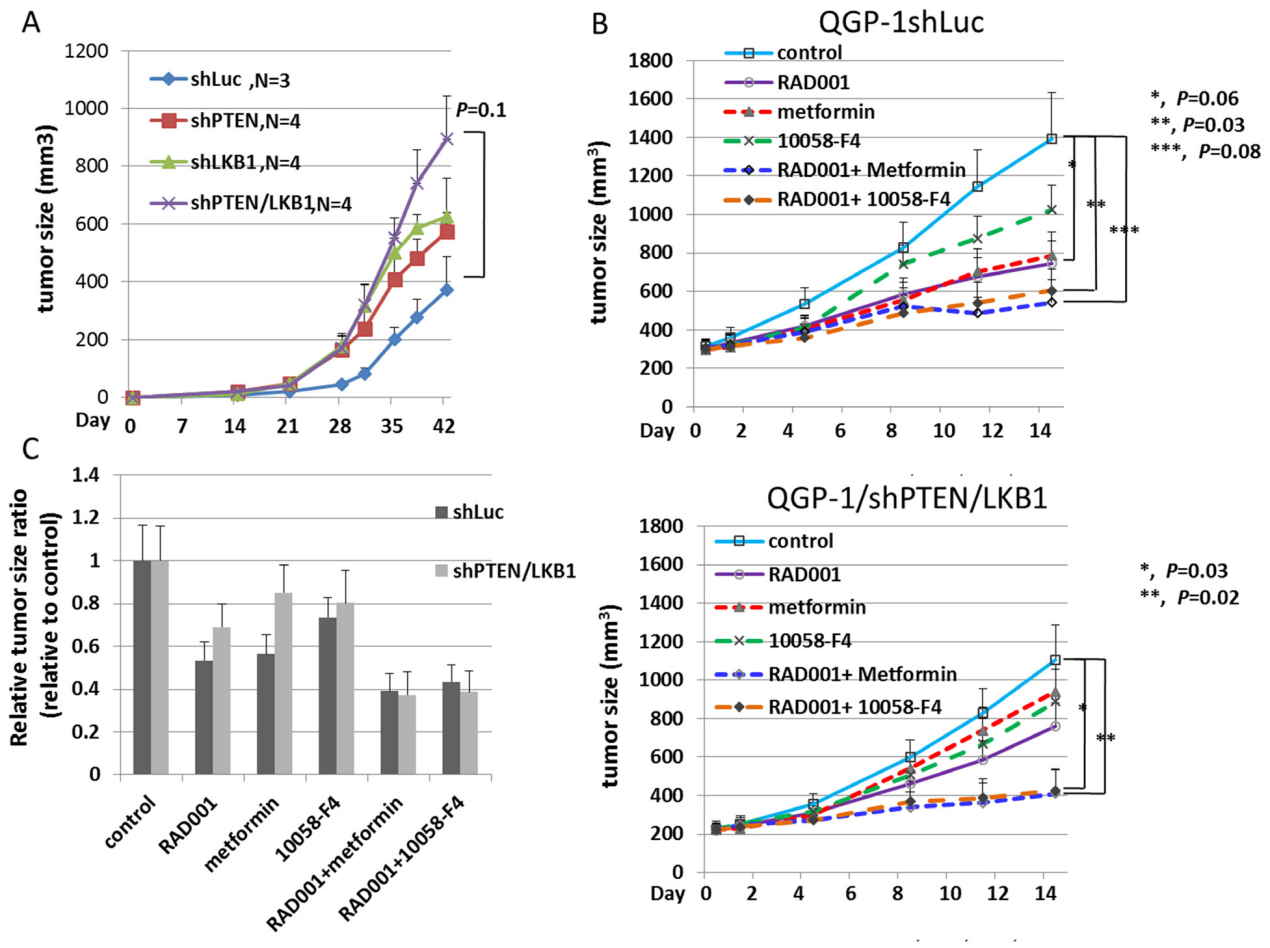
The tumor growth curves of QGP-1 xenograft mice and anti-tumor response of QGP-1 xenograft mice treated with RAD001, metformin, 10058-F4 and combination of RAD001 with metformin or 10058-F4 (**A**) The tumor growth curves of QGP-1/shLuc, QGP-1/shPTEN, QGP-1/shLKB1 and QGP-1/shPTEN/LKB1 xenograft mice from Day 0 to Day 42. The tumor sizes were represented with mean ± standard error. The *p*-value for the tumor size difference between shLuc and shPTEN/LKB1 was 0.1. (**B**) The tumor growth curves of QGP-1/shLuc (upper) and QGP-1/shPTEN/LKB1 (lower) xenograft mice treated with RAD001 (5mg/Kg), metformin (250mg/Kg), 10058-F4 (30mg/Kg), RAD001 + metformin, or RAD001 + 10058-F4. The tumor size is represented with mean ± standard error. For QGP-1/shLuc mice, ^*^, control vs. RAD001, *P* = 0.06 ; ^**^, control vs. RAD001 + 10058-F4, *P* = 0.03; ^***^, control vs. RAD001 + Metformin, *P* = 0.08. For QGP-1/shPTEN/LKB1 mice, ^*^, control vs. RAD001 + 10058-F4, *P* = 0.03; ^**^, control vs. RAD001 + metformin, *P*=0.02. (**C**) The ratio of tumor size in drug treated group relative to the tumor size in control group.

To evaluate the proliferation of pNET cells *in vivo*, we injected QGP-1/shLuc, QGP-1/shPTEN, QGP-1/shLKB1 and QGP-1/shPTEN/LKB1 cells subcutaneously into NOD-SCID mice and observed tumor growth. There were 3 mice in the QGP-1/shLuc group, and 4 mice each in the other 3 groups. Figure 6A shows that QGP-1/shPTEN/LKB1 mice presented rapid tumor growth. QGP-1/shLKB1 and QGP-1/shPTEN mice showed a slight increase in tumor growth compared to that in QGP-1/shLuc mice, which was similar to the *in vitro* result although not statistically significant. To evaluate if the drug resistance of RAD001 observed in QGP-1/shPTEN/LKB1 cells can be reversed by the addition of metformin or a c-Myc inhibitor *in vivo*, we treated the mice inoculated with QGP-1/shLuc and QGP-1/shPTEN/LKB1 cells with RAD001 alone, metformin alone, 10058-F4 alone, RAD001 + metformin or RAD001+ 10058-F4 for 2 weeks (5 days treatment and 2 days rest per week) and observed tumor growth. There were 8 mice in each treatment group. Figure 6B shows the tumor growth curves of these mice. For QGP-1/shLuc mice, three in the metformin group died by day 11, and 4 mice in the RAD001 + metformin group died by day 14. For QGP-1/shPTEN/LKB1 mice, there was one mouse in the metformin group that died by day 11, and the other mice were all alive by the end of the 2-week treatment. For both QGP-1/shLuc and QGP-1/shPTEN/LKB1 mice, the RAD001 + 10058-F4 and RAD001 + metformin treatments had the best inhibitory effects on tumor growth. QGP-1/shPTEN/LKB1 mice were more resistant to RAD001 compared to QGP-1/shLuc mice, but this difference was not statistically significant. However, the drug resistance was reversed by the addition of metformin or 10058-F4. The relative anti-tumor effect induced by RAD001, metformin, 10058-F4 and combination of the agents is shown in Figure 6C. These results demonstrated that metformin or 10058-F4 may reverse the attenuated sensitivity of QGP-1/shPTEN/LKB1 cells to RAD001 *in vivo* similar to what was observed *in vitro*.

### c-Myc expression in pNET patients

We evaluated the c-Myc expression status in tumor samples from pNET patients by immunohistochemistry. c-Myc staining was present in the nuclei of tumors. The c-Myc expression for two cases is presented in Figure 1. The demographics and expression status of c-Myc of the patients are listed in Supplementary Table 1. Among the 21 pNET patients, 17 patients had high expression of c-Myc, which was not only found in the patients with low expression of PTEN and/or LKB1 but also in those with high expression of PTEN and LKB1. No association of c-Myc expression was found with the sex, age, grade, stage or survival of the patients (Supplementary Table 2), and no association of c-Myc with PTEN and LKB1 was found (Supplementary Table 3). These results suggested that regulation of c-Myc occurs through PTEN/LKB1-dependent and -independent mechanisms. Among the 21 pNET patients, there were 5 patients treated with everolimus. The patient characteristics, expression levels of PTEN, LKB1 and c-Myc as well as the response of these 5 patients to everolimus are listed in Supplementary Table 4. Two patients with 3+ expression of c-Myc were not responsive to everolimus. The statistical analysis for the correlation of PTEN, LKB1 and c-Myc with drug sensitivity was not performed due to limited case numbers.

## DISCUSSION

In this study, we found that 48% and 24% of the 21 pNET patients had a low expression of PTEN and LKB1, respectively, and we also found that 81% of the 21 pNET patients had a high expression of c-Myc. We demonstrated that the deficiency of PTEN and LKB1 synergistically promoted the proliferation of pNET cells and attenuated the sensitivity of pNET cells to mTOR inhibitors via up-regulation of AKT/mTOR/c-Myc. Targeting c-Myc or modulation of the mTOR pathway by an AMPK activator reversed the attenuated sensitivity of pNET cells with PTEN and LKB1 loss to mTOR inhibitors *in vitro* and *in vivo*.

PTEN deficiency induces tumor progression or invasion via different pathways, including the PI3K/AKT/mTOR pathway, in various cancers [17, 18]. Loss of LKB1 activates the mTOR pathway and promotes cell growth, survival and tumorigenesis in acute myeloid leukemia, squamous cell carcinoma of skin, and squamous cell carcinoma of lung via LKB1/AMPK regulation [19–21]. Synergistic loss of PTEN and LKB1 leads to the development of various types of cancers in mouse models [13, 22–24]. In our study, we demonstrated enhanced proliferation of pNET cell lines resulting from PTEN and/or LKB1 loss via activation of the AKT/mTOR pathway and up-regulation of c-Myc.

c-Myc is an oncogenic transcription factor and plays a major role in the promotion of ribosomal RNA biosynthesis, cell growth and cell proliferation [25]. c-Myc also modulates resistance to chemotherapy or mTOR inhibitors in leukemia stem cells or breast cancers [26, 27]. c-Myc can be regulated by the PTEN, LKB1, AKT and mTOR pathway at transcriptional or posttranscriptional levels in cancer cells [28–31]. c-Myc is considered the downstream target of the AKT/mTOR pathway in pNET cells because c-Myc expression in QGP-1/shPTEN cells was reduced by AKT knockdown or treatment with RAD001, a mTOR inhibitor. In contrast, c-Myc overexpression in QGP-1 cells promoted cell growth and induced activation of AKT and 4EBP1. Downregulation of c-Myc in QGP-1/shPTEN/LKB1 cells reduced the phosphorylation of AKT, mTOR, S6K and 4EBP1. These results indicated that c-Myc is not only downstream of AKT/mTOR but can also back regulate the AKT/mTOR pathway in pNET cells. According to Guo et al., c-Myc up-regulates miR-26a, which suppresses PTEN expression in glioblastoma multiforme cells [32]. In our study, the regulation of AKT by c-Myc in QGP-1 cells occurred through negative regulation of PTEN by c-Myc as shown in Figure 4C and Supplementary Figure 2. In addition, 81% of pNET patients, which included those with high expression of PTEN and LKB1, had high expression of c-Myc in their tumor tissues, suggesting that the up-regulation of c-Myc may be attributed to the PTEN/LKB1-dependent and -independent regulation in pNET. Activation of the AKT/mTOR pathway by c-Myc overexpression via a PTEN/LKB1-independent mechanism may overcome the suppressive effect of PTEN/LKB1 on the AKT/mTOR/c-Myc axis and explain the high expression of c-Myc in pNET patients with high expression of PTEN and LKB1. c-Myc associates with the resistance to mTOR inhibitors in breast and colorectal cancers [27, 33]. Our study was consistent with these previously reported results in that up-regulated c-Myc was associated with resistance to everolimus in pNET cells. However, this correlation was not defined in the patients in our study. Only 5 of 21 pNET patients received everolimus treatment in our study, and all of them had high expression (2+ or 3+) of c-Myc. We found that 2 patients with strong expression (3+) of c-Myc were not responsive to everolimus, while 3 patients with moderate expression (2+) of c-Myc were responsive to everolimus. Because the case number was too limited, we were unable to correlate the association of c-Myc expression with everolimus sensitivity.

**Figure 7:**
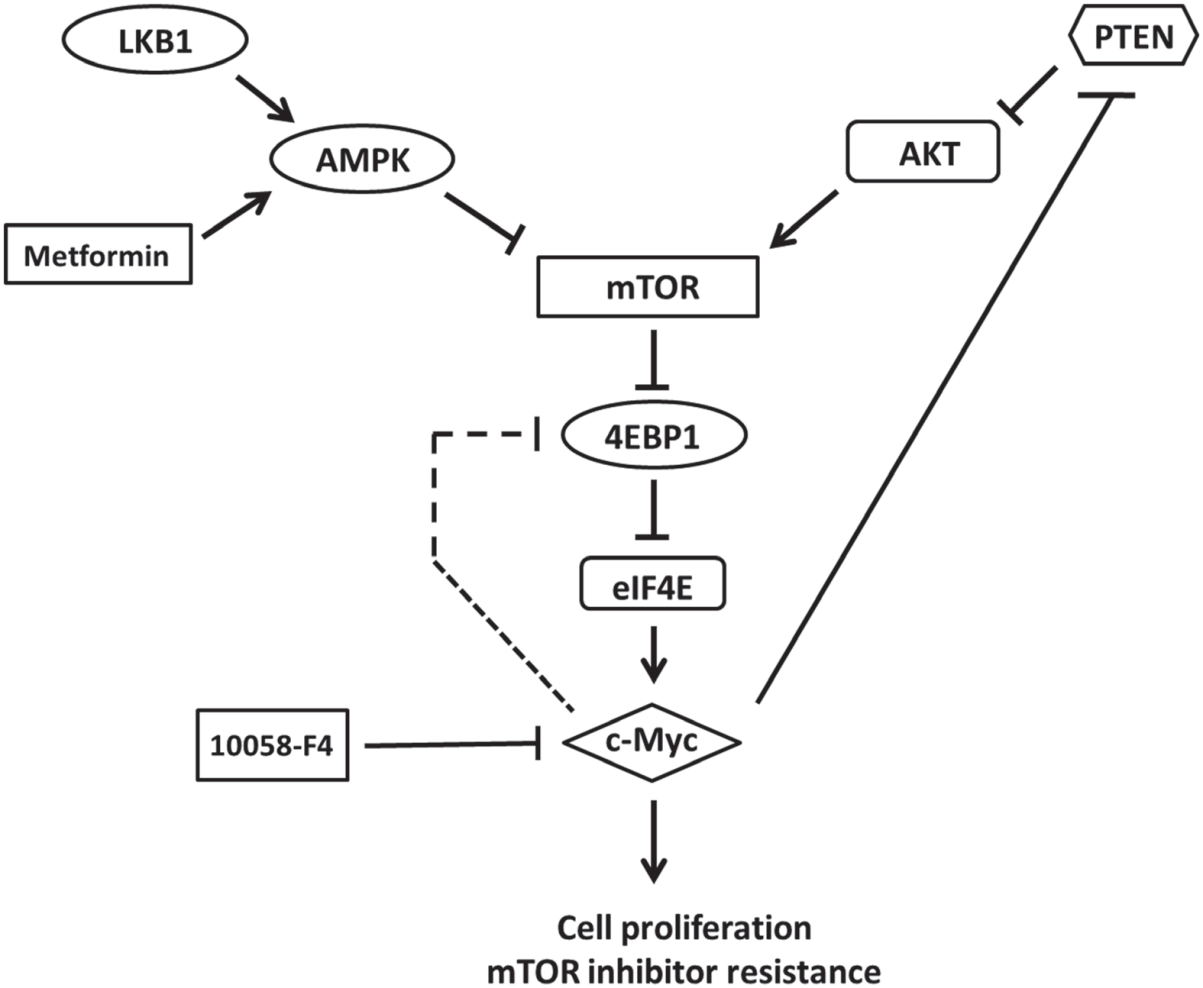
The putative model for the regulation of AKT/mTOR/c-Myc axis by PTEN and LKB1 in pNET PTEN and LKB1 can inhibit mTOR and its downstream effector, 4EBP1 and subsequent activation of c-Myc via inhibiting AKT activation and activating AMPK, respectively. However, c-Myc can activate mTOR through negatively back-regulating PTEN with activation of AKT or feedback activation of 4EBP1 directly and results in activation of c-Myc further. Metformin, an AMPK activator, can inhibit mTOR, 4EBP1 and c-Myc via activation of AMPK. 10058-F4, a c-Myc inhibitor, can also inhibit c-Myc and mTOR and 4EBP1 via inhibiting feedback regulation of mTOR and 4EBP1 by c-Myc.

In mouse endometrial and bladder cancers, loss of PTEN and LKB1 leads to activation of the AKT/mTOR pathway and results in tumors sensitivity to PI3K and mTOR inhibition. However, different from the two mouse models, our results showed that PTEN and LKB1 loss induced attenuated sensitivity of pNET cells to mTOR inhibitors. Therefore, PTEN and LKB1 loss not only confers proliferative advantage of pNET cells but also attenuates the sensitivity of pNET cells to mTOR inhibitors via up-regulating the AKT/mTOR/c-Myc axis. The putative model for the regulation of AKT/mTOR/c-Myc by PTEN and LKB1 and the back regulation of PTEN/AKT/mTOR by c-Myc is shown in Figure 7.

c-Myc is thought to mitigate the response to rapamycin in prostate cancer through up-regulation of 4EBP1 [34]. In our study, RAD001 suppressed phosphorylation of 4EBP1 in QGP-1/shPTEN/LKB1 and QGP-1/shLuc cells, but it had a lesser effect in inhibiting the proliferation of QGP-1/shPTEN/LKB1 cells than that of QGP-1/shLuc cells, suggesting the exclusion of the mTOR downstream factor, 4EBP1, as the key factor to induce drug resistance in QGP-1/shPTEN/LKB1 cells. Furthermore, combination of the c-Myc inhibitor, 10058-F4, with RAD001 synergistically suppressed c-Myc expression in QGP-1/shPTEN/LKB1 cells and reversed sensitivity of QGP-1/shPTEN/LKB1 cells to RAD001. These results further confirmed the association of c-Myc and resistance of pNET cells to mTOR inhibitors. Therefore, targeting c-Myc is a potential therapeutic approach for the treatment of pNET.

PTEN loss is noted in many cancer types and is associated with poor clinical outcome [35, 36]. Altered expression of PTEN is also present in pulmonary NETs [37]. The percentage of low PTEN expression in pNET patients varies and ranges from 10 to 50% with a trend for an association with tumor grading and survival [4, 6, 11, 38, 39]. In our study, low expression of PTEN was identified in 10 patients (47.6%). The percentage for low PTEN expression in pNET was close to that reported by Missiaglia et al. [4] and Qian et al. [6]. Due to a limited case number, we did not find significant correlation between grade, stage or survival of the patients with their PTEN expression status. Low expression of LKB1 (0 to < 20%) has been noted in 45/53 (84.9%) high grade neuroendocrine carcinomas of the lung but only in 3/15 (20%) pulmonary carcinoids [16]. In this study, we found a similar percentage of low expression of LKB1 in pNET as that in pulmonary NET. Although we could not clarify the effect of LKB1 on pNET due to the limited case number in our study, the association between aberrant LKB1 with or without PTEN deficiency and clinical outcome of pNET patients is worthy of further investigation.

c-Myc overexpression is commonly found in Merkle cell carcinoma (MCC), an aggressive neuroendocrine tumor of skin. Inhibition of c-Myc by shRNA or the bromodomain and extra-terminal (BET) inhibitor in MCC cells induces cell cycle arrest and inhibits tumor growth in MCC xenograft mice [40]. Inhibition of cell proliferation and induction of cell cycle arrest has been demonstrated in human pNET cell lines when exposed to the BET inhibitor, CPI203, via targeting c-Myc [41]. In our study, we used 10058-F4 to inhibit c-Myc by interfering with the interaction of c-Myc and MAX, which is required for transactivation of c-Myc without the need for the up-regulation of c-Myc [42]. The combination of 10058-F4 and RAD0001 enhanced the anti-proliferative effect of RAD001 against QGP-1 cells both *in vitro* and *in vivo*, which reinforced the importance of c-Myc activation for cell growth and survival of pNET.

Metformin is a widely administered anti-diabetic therapeutic agent. Metformin inhibits the cell growth of various cancers alone or in combination with other therapeutic agents via LKB1-dependent or LKB1-independent activation of AMPK and subsequent suppression of mTOR and its downstream effectors [43–47]. Metformin can suppress mTORC1 and its downstream proteins in pNET, midgut NET, and pulmonary NET cell lines as well as inhibit cell proliferation of all three cell lines [48]. Currently, a phase II study (MetNET-1 trial, NCT 02294006) to evaluate the activity and safety of everolimus (RAD001) in combination with octreotide LAR and metformin in advanced pNET patients is ongoing [49]. In our study, metformin combined with RAD001 exerted an anti-proliferative effect against QGP-1/shPTEN/LKB1 cells via suppression of mTOR, S6K, 4EBP1 and c-Myc. The potent anti-tumor effect of metformin combined with RAD001 was also observed in our animal study, although higher mortality was noted in the metformin-treated groups. These results suggested the importance of selecting the optimal dose of metformin and supported the potential role of metformin in combination with RAD001 for pNET treatment.

In conclusion, we identified aberrant expression of PTEN, LKB1 and c-Myc in pNET patients. Loss of PTEN and LKB1 or activation of c-Myc promoted the proliferative advantage of pNET and attenuated the sensitivity of pNET cells to mTOR inhibitors via activation of the AKT/mTOR/c-Myc axis in pNET. Combination of metformin or 10058-F4 with RAD001 enhanced the anti-proliferative effect of RAD001 against pNET cells *in vitro* and *in vivo*. These results suggested that c-Myc is a potential therapeutic target in addition to mTOR for the treatment of pNET.

## MATERIALS AND METHODS

### Cell lines, plasmids and reagents

QGP-1, a human pNET cell line, was purchased from the Japanese Collection of Research Bioresources *(JCRB*, Tokyo, Japan). NIT-1, a mouse pNET cell line, was purchased from the Bioresource Collection and Research Center (BCRC, Hsinchu, Taiwan). The passages of the two cell lines used in this study were less than 15. We sent the QGP-1 cell line to the Center for Genomic Medicine of National Cheng Kung University for genotyping in June 2016, and the result showed the same STR PCR DNA profile as those in the JCRB database. QGP-1 and NIT-1 cells were cultured in RPMI-1640 (HyClone, South Logan, Utah, USA) medium and F12-Kaighn's (Gibco, Grand Island, NY, USA) medium, respectively, containing 10% fetal calf serum and antibiotics. PTEN and c-Myc expression plasmids were purchased from Addgene (Cambridge, MA, USA). shRNAs targeting PTEN, LKB1, AKT and c-Myc were obtained from the National RNAi Core Facility of Academic Sinica (Taipei, Taiwan). Rapamycin and RAD001 were purchased from LC Laboratories (Boston, MA, USA) and Selleckchem (Houston, TX, USA), respectively. Metformin was purchased from TOCRIS (Bristol, UK), and 10058-F4 was purchased from Calbiochem (San Diego, CA, USA).

### Stable cell establishment

Stable gene overexpression or knockdown in QGP-1 and NIT-1 cells was conducted by the lentiviral infection system according to Addgene instructions. We replaced virus medium with fresh medium 24 hours after lentiviral infection, and the cells were treated with puromycin to select infected cells. Stable cells were confirmed by western blot analysis.

### Western blot analysis

Lentivirus-infected or drug-treated cells were harvested in lysis buffer. An equal amount of proteins was subjected to SDS-PAGE. Proteins were transferred onto PVDF membranes, and the blots were incubated with the following different primary antibodies: PTEN and LKB1 from Santa Cruz Biotechnology (Dallas, USA); AKT, p-AKT(S473), mTOR, p-mTOR (S2448), S6K, p-S6K (T389), 4EBP1, and p-4EBP1 (T70) from Cell Signaling (Danvers, MA, USA); and c-Myc and Actin from Abcam (Cambridge, MA, USA).Enhanced chemiluminescence reagents were used to depict the protein bands on the membrane, and the bands were visualized by an UVP biospectrum image system (Upland, CA, USA). Representative images are shown from duplicate or triplicate experiments.

### Methylene blue colorimetric and MTT assays

Proliferation of cells with knockdown or overexpression of indicated genes and survival rate of the cells treated with or without mTOR, c-Myc inhibitors or AMPK activator were analyzed. Briefly, 20,000 cells were seeded in 24-well culture plates with or without indicated agents for indicated durations. Cell proliferation or survival was measured by methylene blue or MTT assays. For the methylene blue assay, 0.5% methylene blue solution was incubated with adherent cells in microplate for 1 hour and then removed by PBS wash. Finally, 1% sarcosine was added to each well of the microplate to dissolve the methylene blue, and growth curves were determined according to absorbance at 595 nm using a SpectraMax M5 microplate reader (Molecular Devices, Sunnyvale, CA, USA). For the MTT assay, 20,000 cells were seeded in 24-well culture plates with or without indicated agents for indicated durations. MTT was added directly to the culture medium and incubated at 37°C for 4 hours. The medium was then replaced with DMSO, and cell proliferation and survival were determined according the absorbance at 570 nm using a SpectraMax M5 microplate reader. The cell proliferative rate of the cells with indicated gene aberrations was 1 at day 0, and the proliferative rate of the cells from day 1 to day 5 was calculated by dividing the absorbance at 595 nm each day by the absorbance at 595 nm at day 0. The survival rate of cells not treated with mTOR, c-Myc inhibitor or AMPK activator was presented as 1 (100%), and the survival rate of the cells treated with the indicated drugs was calculated by dividing the absorbance at 595 or 570 nm of cells exposed to drugs by the absorbance at 595 or 570 nm of cells without drug exposure. Each data point was in triplicate, and the result is presented as the mean ± standard error.

### Immunohistochemistry and scoring

Tumor tissues were obtained from pNET patients diagnosed at the National Cheng Kung University Hospital from 2006 to 2015. This study was approved by the Institutional Review Board (A-BR-102-025), and informed consent was obtained from all patients. Immunostaining was performed with a Leica Bond-Max automatic immunostainer (Leica, Bannockburn, IL) following 25 minutes of incubation at room temperature in Bond™ Epitope Retrieval Solution 1 (Leica Biosystems, Catalog No. AR9961). Slides were incubated with a 1:200 dilution of anti-LKB1 antibody (Santa Cruz Biotechnology), a 1:200 dilution of anti-PTEN antibody (6H2.1, Genemed, South San Francisco, CA, USA), or a 1:100 dilution of anti-c-Myc antibody (aa386-435, Abgent, San Diego, CA, USA) for 1 hour at room temperature. Paraffin-embedded sections of human breast cancer cells were included as positive controls for PTEN. Negative controls replaced the primary antibody with PBS. The expression of LKB1, PTEN, and c-Myc was rated semiquantitatively based on the staining intensity and staining percentage. The staining intensity was scored as “-,” “1+,” “2+,” and “3+” for “Negative,” “Weak,” “Moderate” and “Strong” staining, respectively.

### *In vivo* study

NOD-SCID (6 to 8 weeks old) male mice were obtained from LASCO (Taipei, Taiwan) and housed under specific pathogen-free conditions according to the guidelines of the Animal Care Committee at the National Health Research Institutes, Taiwan. QGP-1 (shLuc, shPTEN, shLKB1 and shPTEN/LKB1) cells (1×10^7^) mixed with Matrigel (BD Biosciences, San Jose, CA, USA) in 0.1 mL were injected subcutaneously into each mouse. Tumor volume was measured by caliper measurements and calculated as length (mm) x width^2^ (mm) x (π/6) [50]. The mice (QGP-1/shLuc or QGP-1/shPTEN/LKB1) were orally or intraperitoneally injected with the indicated dose of RAD001 (5mg/kg), metformin (250mg/kg) or 10058-F4 (30mg/kg) either alone or in combination for 2 weeks after tumors developed to approximately 200 to 300 mm^3^. Tumor volumes were measured twice a week from the initiation of treatment to 2 weeks.

### Statistical analysis

The difference in the proliferative rate between different clones of cells was analyzed by *t*-test. The difference in the survival rate for the cells treated with various drugs compared to the control was analyzed by the *t*-test using EXCEL (Microsoft, Redmond, WA, USA). The correlation between clinical data and immunohistochemical staining for PTEN, LKB1, and c-Myc expression was analyzed by the Fisher's exact test using SAS software (SASInstitute Inc, Cary, NC, USA). The comparison for the differences in tumor size in the animal study was analyzed by the Wilcoxon rank-sum test using SAS (SASInstitute Inc, Cary, NC, USA).

## SUPPLEMENTARY MATERIALS FIGURES AND TABLES



## References

[1] Yao JC, Hassan M, Phan A, Dagohoy C, Leary C, Mares JE, Abdalla EK, Fleming JB, Vauthey JN, Rashid A, Evans DB (2008). One hundred years after “carcinoid”: epidemiology of and prognostic factors for neuroendocrine tumors in 35,825 cases in the United States. J Clin Oncol.

[2] Hauso O, Gustafsson BI, Kidd M, Waldum HL, Drozdov I, Chan AK, Modlin IM (2008). Neuroendocrine tumor epidemiology: contrasting Norway and North America. Cancer.

[3] Tsai HJ, Wu CC, Tsai CR, Lin SF, Chen LT, Chang JS (2013). The Epidemiology of Neuroendocrine Tumors in Taiwan: a Nation-wide Registry-based Study. PLoS One.

[4] Missiaglia E, Dalai I, Barbi S, Beghelli S, Falconi M, della Peruta M, Piemonti L, Capurso G, Di Florio A, delle Fave G, Pederzoli P, Croce CM, Scarpa A (2010). Pancreatic endocrine tumors: expression profiling evidences a role for AKT-mTOR pathway. J Clin Oncol.

[5] Zhou CF, Ji J, Yuan F, Shi M, Zhang J, Liu BY, Zhu ZG (2011). mTOR activation in well-differentiated pancreatic neuroendocrine tumors: a retrospective study on 34 cases. Hepatogastroenterology.

[6] Qian ZR, Ter-Minassian M, Chan JA, Imamura Y, Hooshmand SM, Kuchiba A, Morikawa T, Brais LK, Daskalova A, Heafield R, Lin X, Christiani DC, Fuchs CS (2013). Prognostic significance of MTOR pathway component expression in neuroendocrine tumors. J Clin Oncol.

[7] Yao JC, Shah MH, Ito T, Bohas CL, Wolin EM, Van Cutsem E, Hobday TJ, Okusaka T, Capdevila J, de Vries EG, Tomassetti P, Pavel ME, Hoosen S (2011). RAD001 in Advanced Neuroendocrine Tumors, Third Trial (RADIANT-3) Study Group. Everolimus for advanced pancreatic neuroendocrine tumors. N Engl J Med.

[8] Li J, Yen C, Liaw D, Podsypanina K, Bose S, Wang SI, Puc J, Miliaresis C, Rodgers L, McCombie R, Bigner SH, Giovanella BC, Ittmann M (1997). PTEN, a putative protein tyrosine phosphatase gene mutated in human brain, breast, and prostate cancer. Science.

[9] Steck PA, Pershouse MA, Jasser SA, Yung WK, Lin H, Ligon AH, Langford LA, Baumgard ML, Hattier T, Davis T, Frye C, Hu R, Swedlund B (1997). Identification of a candidate tumour suppressor gene, MMAC1, at chromosome 10q23.3 that is mutated in multiple advanced cancers. Nat Genet.

[10] Song AM, Salmena L, Pandolfi PP (2012). The functions and regulation of the PTEN tumour suppressor. Nat Rev Mol Cell Biol.

[11] Estrella JS, Broaddus RR, Mathews A, Milton DR, Yao JC, Wang H, Rashid A (2014). Progesterone receptor and PTEN expression predict survival in patients with low- and intermediate-grade pancreatic neuroendocrine tumors. Arch Pathol Lab Med.

[12] DeGraffenried LA, Fulcher L, Friedrichs WE, Grünwald V, Ray RB, Hidalgo M (2004). Reduced PTEN expression in breast cancer cells confers susceptibility to inhibitors of the PI3 kinase/Akt pathway. Ann Oncol.

[13] Cheng H, Liu P, Zhang F, Xu E, Symonds L, Ohlson CE, Bronson RT, Maira SM, Di Tomaso E, Li J, Myers AP, Cantley LC, Mills GB, Zhao JJ (2013). A genetic mouse model of invasive endometrial cancer driven by concurrent loss of Pten and Lkb1 is highly responsive to mTOR inhibition. Cancer Res.

[14] Patel M, Gomez NC, McFadden AW, Moats-Staats BM, Wu S, Rojas A, Sapp T, Simon JM, Smith SV, Kaiser-Rogers K, Davis IJ (2014). PTEN deficiency mediates a reciprocal response to IGFI and mTOR inhibition. Mol Cancer Res.

[15] Shackelford DB, Shaw RJ (2009). The LKB1-AMPK pathway: metabolism and growth control in tumour suppression. Nat Rev Cancer.

[16] Amin RM, Hiroshima K, Iyoda A, Hoshi K, Honma K, Kuroki M, Kokubo T, Fujisawa T, Miyagi Y, Nakatani Y (2008). LKB1 protein expression in neuroendocrine tumors of the lung. Pathol Int.

[17] Asano T, Yao Y, Zhu J, Li D, Abbruzzese JL, Reddy SA (2004). The PI3-kinase/Akt signaling pathway is activated due to aberrant Pten expression and targets transcription factors NF-kB and c-Myc in pancreatic cancer cells. Oncogene.

[18] Guo Y, Chang H, Li J, Xu XY, Shen L, Yu ZB, Liu WC (2015). Thymosin alpha 1 suppresses proliferation and induces apoptosis in breast cancer cells through PTEN-mediated inhibition of PI3K/Akt/mTOR signaling pathway. Apoptosis.

[19] Shaw RJ, Bardeesy N, Manning BD, Lopez L, Kosmatka M, DePinho RA, Cantley LC (2004). The LKB1 tumor suppressor negatively regulates mTOR signaling. Cancer Cell.

[20] Gurumurthy S, Hezel AF, Berger JH, Berger JH, Bosenberg MW, Bardeesy N (2008). LKB1 deficiency sensitizes mice to carcinogen-induced tumorigenesis. Cancer Res.

[21] Green AS, Chapuis N, Maciel TT, Willems L, Lambert M, Arnoult C, Boyer O, Bardet V, Park S, Foretz M, Viollet B, Ifrah N, Dreyfus F (2010). The LKB1/AMPK signaling pathway has tumor suppressor activity in acute myeloid leukemia through the repression of mTOR-dependent oncogenic mRNA translation. Blood.

[22] Huang X, Wullschleger S, Shpiro N, McGuire VA, Sakamoto K, Woods YL, McBurnie W, Fleming S, Alessi DR (2008). Important role of the LKB1-AMPK pathway in suppressing tumorigenesis in PTEN-deficient mice. Biochem J.

[23] Shorning BY, Griffiths D, Clarke AR (2011). Lkb1 and Pten synergise to suppress mTOR-mediated tumorigenesis and epithelial-mesenchymal transition in the mouse bladder. PLoS One.

[24] Xu C, Fillmore CM, Koyama S, Wu H, Zhao Y, Chen Z, Herter-Sprie GS, Akbay EA, Tchaicha JH, Altabef A, Reibel JB, Walton Z, Ji H (2014). Loss of Lkb1 and Pten leads to lung squamous cell carcinoma with elevated PD-L1 expression. Cancer Cell.

[25] Pelegaris S, Khan M, Evan G (2002). c-Myc: more than just a matter of life and death. Nat Rev Cancer.

[26] Zhang Y, Chen HX, Zhou SY, Wang SX, Zheng K, Xu DD, Liu YT, Wang XY, Wang X, Yan HZ, Zhang L, Liu QY, Chen WQ, Wang YF (2015). Sp1 and c-Myc modulate drug resistance of leukemia stem cells by regulating survivin expression through the ERK-MSK MAPK signaling pathway. Molecular Cancer.

[27] Bihani T, Ezell SA, Ladd B, Grosskurth SE, Mazzola AM, Pietras M, Reimer C, Zinda M, Fawell S, D’Cruz CM (2015). Resistance to everolimus driven by epigenetic regulation of MYC in ER+ breast cancers. Oncotarget.

[28] Ghosh AK, Grigorieva I, Steele R, Hoover RG, Ray RB (1999). PTEN transcriptionally modulates c-myc gene expression in human breast carcinoma cells and is involved in cell growth regulation. Gene.

[29] Yeh ES, Belka GK, Vernon AE, Chen CC, Jung JJ, Chodosh LA (2013). Hunk negatively regulates c-myc to promote Akt-mediated cell survival and mammary tumorigenesis induced by loss of Pten. Proc Natl Acad Sci U S A.

[30] Csibi A, Lee G, Yoon SO, Tong H, Ilter D, Elia I, Fendt SM, Roberts TM, Blenis J (2014). The MTORC1/S6K1 pathway regulates glutamine metabolism through the eIF4B-dependent control of c-Myc translation. Curr Biol.

[31] Liang X, Nan KJ, Li ZL, Xu QZ (2009). Overexpression of the LKB1 gene inhibits lung carcinoma cell proliferation partly through degradation of c-myc protein. Oncol Rep.

[32] Guo P, Nie Q, Lan J, Ge J, Qiu Y, Mao Q (2013). C-Myc negatively controls the tumor suppressor PTEN by upregulating miR-26a in glioblastoma multiforme cells. Biochem Biophys Res Commun.

[33] Tan J, Lee PL, Li Z, Jiang X, Lim YC, Hooi SC, Yu Q (2010). B55b-associated PP2A complex controls PDK-1 directed Myc signaling and modulates rapamycin sensitivity in colorectal cancer. Cancer Cell.

[34] Balakumaran BS, Porrello A, Hsu DS, Glover W, Foye A, Leung JY, Sullivan BA, Hahn WC, Loda M, Febbo PG (2009). Myc activity mitigates response to rapamycin in prostate cancer through eukaryotic initiation factor 4E-binding protein 1-mediated inhibition of autophagy. Cancer Res.

[35] McCall P, Witton CJ, Grimsley S, Nielsen KV, Edwards J (2008). Is PTEN loss associated with clinical outcome measures in human prostate cancer?. Br J Cancer.

[36] Colakoglu T, Yildirim S, Kayaselcuk F, Nursal TZ, Ezer A, Noyan T, Karakayali H, Haberal M (2008). Clinicopathological significance of PTEN loss and the phosphoinositide 3-kinase/Akt pathway in sporadic colorectal neoplasms: is PTEN loss predictor of local recurrence?. Am J Surg.

[37] Lee HW, Ha SY, Roh MS (2014). Altered expression of PTEN and its major regulator microRNA-21 in pulmonary neuroendocrine tumors. Korean J Pathol.

[38] Han X, Ji Y, Zhao J, Xu X, Lou W (2013). Expression of PTEN and mTOR in pancreatic neuroendocrine tumors. Tumor Biol.

[39] Krausch M, Raffel A, Anlauf M, Schott M, Willenberg H, Lehwald N, Hafner D, Cupisti K, Eisenberger CF, Knoefel WT (2011). Loss of PTEN expression in neuroendocrine pancreatic tumors. Horm Metab Res.

[40] Shao Q, Kannan A, Lin Z, Stack BC, Suen JY, Gao L (2014). BET protein inhibitor JQ1 attenuates Myc-amplified MCC tumor growth in vivo. Cancer Res.

[41] Wong C, Laddha SV, Tang L, Vosburgh E, Levine AJ, Normant E, Sandy P, Harris CR, Chan CS, Xu EY (2014). The bromodomain and extra-terminal inhibitor CPI203 enhances the antiproliferative effects of rapamycin on human neuroendocrine tumors. Cell Death Dis.

[42] Huang MJ, Cheng YC, Liu CR, Lin S, Liu HE (2006). A small-molecular c-Myc inhibitor, 10058-F4, induces cell0cycle arrest, apoptosis, and myeloid differentiation of human acute myeloid leukemia. Exp Hematol.

[43] Guo Q, Liu Z, Jiang L, Liu M, Ma J, Yang C, Han L, Nan K, Liang X (2016). Metformin inhibits growth of human non-small cell lung cancer cells via liver kinase B-1-independent activation of adenosine monophosphate-activated protein kinase. Mol Med Rep.

[44] Della Corte CM, Ciaramella V, Di Mauro C, Castellone MD, Papaccio F, Fasano M, Sasso FC, Martinelli E, Troiani T, De Vita F, Orditura M, Bianco R, Ciardiello F, Morgillo F (2016). Metformin increases antitumor activity of MEK inhibitors through GLI1 downregulation in LKB1 positive human NSCLC cancer cells. Oncotarget.

[45] Groenendijk FH, Mellema WW, van der Burg E, Schut E, Hauptmann M, Horlings HM, Willems SM, van den Heuvel MM, Jonkers J, Smit EF, Bernards R (2015). Sorafenib synergizes with metformin in NSCLC through AMPK pathway activation. Int J Cancer.

[46] Dowling RJ, Zakikhani M, Fantus IG, Pollak M, Sonenberg N (2007). Metformin inhibits mammalian target of rapamycin-dependent translation initiation in breast cancer cells. Cancer Res.

[47] Morgillo F, Sasso FC, Della Corte CM, Vitagliano D, D’Aiuto E, Troiani T, Martinelli E, De Vita F, Orditura M, De Palma R, Ciardiello F (2013). Synergistic effects of metformin treatment in combination with gefetinib, a selective EGFR tyrosine kinase inhibitor, in LKB1 wild-type NSCLC cell lines. Clin Cancer Res.

[48] Vlotides G, Tanyeri A, Spampatti M, Zitzmann K, Chourdakis M, Spttl C, Maurer J, Nölting S, Göke B, Auernhammer CJ (2014). Anticancer effects of metformin on neuroendocrine tumor cells in vitro. Hormones (Athens).

[49] Pusceddu S, de Braud F, Concas L, Bregant C, Leuzzi L, Formisano B, Buzzoni R (2014). Rationale and protocol of the MetNET-1 trial, a prospective, single center, phase II study to evaluate the activity and safety of everolimus in combination with octreotide LAR and metformin in patients with advanced pancreatic neuroendocrine tumors. Tumori.

[50] Grbovic OM, Basso AD, Sawai A, Ye Q, Friedlander P, Solit D, Rosen N (2006). V600E B-Raf requires the Hsp90 chaperone for stability and is degraded in response to Hsp90 inhibitors. Proc Natl Acad Sci USA.

