# Supplementary material for: The regulatory role of aberrant Phosphatase and Tensin Homologue and Liver Kinase B1 on AKT/mTOR/c-Myc axis in pancreatic neuroendocrine tumors
Source: Oncotarget. 2017 Sep 16;8(58):98068–83. doi: 10.18632/oncotarget.20956 (PMC5716714; doi:10.18632/oncotarget.20956)
Supplement: Supplementary file 1 [file oncotarget-08-98068-s001.pdf]

The regulatory role of aberrant Phosphatase and Tensin Homologue and Liver Kinase B1 on AKT/mTOR/c-Myc axis in pancreatic neuroendocrine tumors

SUPPLEMENTARY MATERIALS

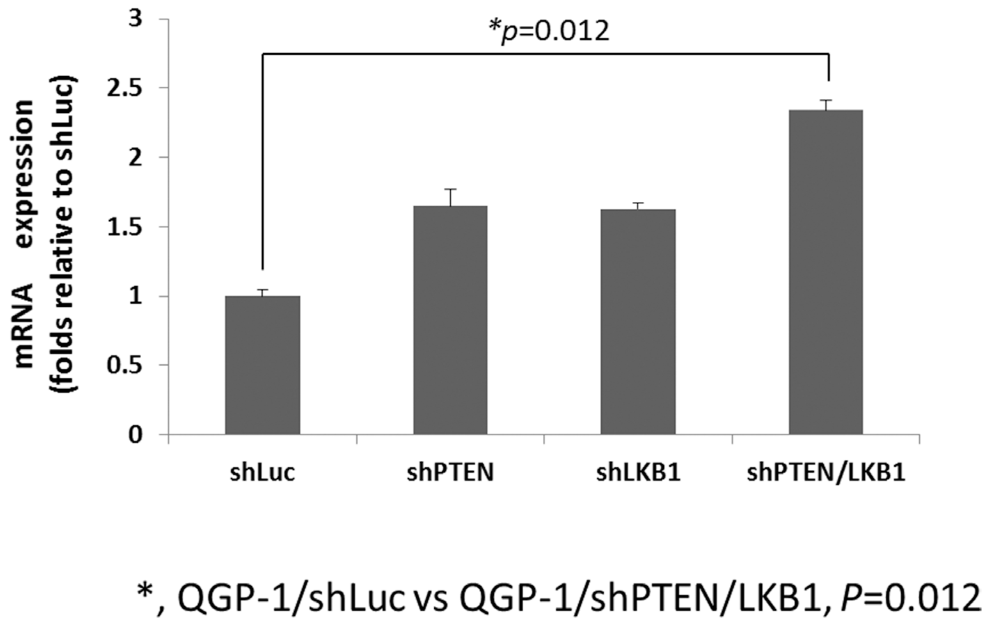

Supplementary Figure 1: The mRNA expression of QGP-1/shLuc, QGP-1/shPTEN, QGP-1 shlKB1 and QGP-1/shPTEN/LKB1.

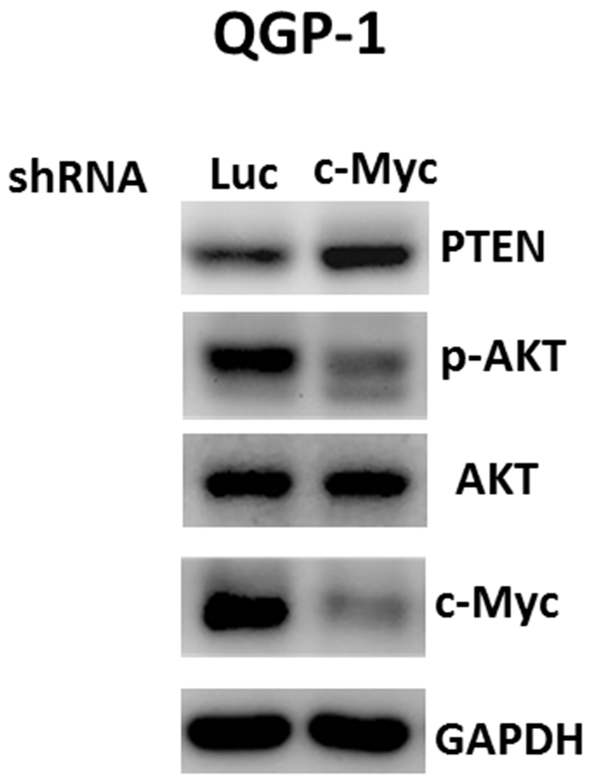

Supplementary Figure 2: The Protein expression of PTEN, AKT, c-Myc and phosphorylated AKT in QGP-1 cells with or without knockdown of c-Myc

Supplementary Table 1: The demographics and expression status of PTEN, LKB1, and c-Myc in pNET patients

Case No.	Sex	Age	Grade	Stage	PTEN	LKB1	c-Myc	Survival
1	F	80	2	IB	C*, 1+	C*, 1+	N**, 2+	alive
2	M	53	1	IA	C, 1+	C, 2+	N, 2+	dead
3	M	45	2	IV	C, 1+	C, 2+	N, 2+	alive
4	M	67	1	IA	C, 1+	C, 2+	N, 2+	alive
5	M	52	2	IV	C, 2+	C, 3+	N, 2+	alive
6	M	54	1	IV	C, 2+	C, 2+	N, 2+	alive
7	F	47	1	IA	C, 1+	C, 1+	N, 1+	alive
8	M	71	1	IIB	C, 1+	C, 2+	N, 2+	alive
9	M	42	2	IV	Negative	C, 1+	N, 2+	alive
10	M	34	2	IV	C, 1+	C, 2+	N, 3+	alive
11	M	61	2	IV	C, 3+	C, 3+	N, 3+	alive
12	F	62	1	IV	C, 1+	C, 2+	N, 2+	alive
13	M	45	1	IB	C, 2+	C, 3+	N, 1+	alive
14	F	54	1	IB	C, 2+	C, 3+	N, 2+	alive
15	M	48	1	IB	C, 2+	C, 3+	N, 3+	dead
16	F	73	1	IIA	C, 2+	C, 1+	N, 1+	alive
17	M	73	1	IA	C, 1+	C, 1+	N, 3+	alive
18	M	21	2	IA	C, 3+	C, 3+	N, 3+	alive
19	F	62	1	IA	C, 3+	C, 3+	N, 2+	alive
20	F	39	1	IA	C, 3+	C, 3+	N, 2+	alive
21	M	32	2	IV	C, 2+	C, 2+	N, 1+	alive

*C, cytoplasm; **N, nucleus.

Supplementary Table 2: The correlation between PTEN, LKB1, or c-Myc with clinical characteristics

	PTEN			LKB1			c-Myc		
	Low	High		Low	High		Low	High	
	N (%)	*N* (%)	*P**	*N* (%)	*N* (%)	*P**	*N* (%)	*N* (%)	*P**
Sex									
F	3 (30.0)	4 (36.4)	1	3 (60.0)	4 (25.0)	0.28	2 (50.0)	5 (29.4)	0.57
M	7 (70.0)	7 (63.6)		2 (40.0)	12 (70.0)		2 (50.0)	12 (70.6)	
Age									
< 60	5 (50.0)	8 (72.7)	0.39	2 (40.0)	11 (68.8)	0.33	3 (75.0)	10 (58.8)	1
> = 60	5 (50.0)	3 (27.3)		3 (60.0)	5 (31.2)		1 (25.0)	7 (41.2)	
Grade									
I	6 (60.0)	7 (63.6)	1	3 (60.0)	10 (62.5)	1	3 (75.0)	10 (58.8)	1
II	4 (40.0)	4 (36.4)		2 (40.0)	6 (37.5)		1 (25.0)	7 (41.2)	
Stage									
I + II	6 (60.0)	7 (63.6)	1	4 (80.0)	9 (56.3)	0.61	3 (75.0)	10 (58.8)	1
IV	4 (40.0)	4 (36.4)		1 (20.0)	7 (43.7)		1 (25.0)	7 (41.2)	
Survival									
No	1 (10.0)	1 (9.1)	1	0 (0.0)	2 (12.5)	1	0 (0.0)	2 (11.8)	1
Yes	9 (90.0)	10 (90.9)		5 (100.0)	14 (87.5)		4 (100.0)	15 (88.2)	

*The *P* value was generated by Fisher's Exact Test.

Supplementary Table 3: The correlation among PTEN, LKB1 and c-Myc expression in pNETs

	PTEN		
	Low	High	
	N (%)	*N* (%)	*P**
LKB1			
Low	4 (40.0)	1 (9.1)	0.15
High	6 (60.0)	10 (90.9)	
c-Myc			
Low	1 (10.0)	3 (27.3)	0.59
High	9 (90.0)	8 (72.7)	

	LKB1		
	Low	High	
	N (%)	*N* (%)	*P**
c-Myc			
Low	2 (40.0)	2 (12.5)	0.23
High	3 (60.0)	14 (87.5)	

**P* was generated by Fisher's Exact Test

Supplementary Table 4: The demographics, expression status of PTEN, LKB1, and c-Myc and response to everolimus in pNET patients

case No.	sex	age	Grade	stage	PTEN	LKB1	c-Myc	survival	response to everolimus
3	M	45	II	IV	1+	2+	2+	alive	yes
6	M	54	I	IV	2+	2+	2+	alive	yes
10	M	34	II	IV	1+	2+	3+	alive	no
11	M	61	II	IV	3+	3+	3+	alive	no
12	F	62	I	IV	1+	2+	2+	alive	yes
